# The Abbreviated Science Anxiety Scale: Psychometric properties, gender differences and associations with test anxiety, general anxiety and science achievement

**DOI:** 10.1371/journal.pone.0245200

**Published:** 2021-02-12

**Authors:** Ahmed M. Megreya, Denes Szűcs, Ahmed A. Moustafa

**Affiliations:** 1 Department of Psychological Sciences, College of Education, Qatar University, Doha, Qatar; 2 Department of Psychology, Centre for Neuroscience in Education, University of Cambridge, Cambridge, United Kingdom; 3 School of Psychology, Western Sydney University, Sydney, Australia; São Paulo State University (UNESP), BRAZIL

## Abstract

Science anxiety refers to students’ negative emotions about learning science. Across two studies, we investigated the psychometric properties of the newly developed Abbreviated Science Anxiety Scale (ASAS), which was adapted from the modified Abbreviated Math Anxiety Scale (m-AMAS) (Carey E., 2017). Using a sample of students in grades 7 to 10 (*N* = 710), Study 1 reported a two-factor structure of the ASAS (learning science anxiety and science evaluation anxiety) and negative associations between the ASAS factors and science achievement. Study 2 replicated this two-factor model in students in grades 11 and 12 (*N* = 362) and found that students in the “Arts” track were more anxious about science than those in “Sciences” track. Both studies consistently reported positive inter-correlations between the ASAS factors, with good internal reliabilities and modest meaningful associations with test anxiety and general anxiety, suggesting that science anxiety might be a distinct construct. Further, female students had higher science anxiety (especially science evaluation anxiety) than male students, even when test anxiety and general anxiety were considered in models. In summary, the ASAS is a brief, valid, and reliable instrument that can be used to guide and improve science education.

## Introduction

It is well-established that emotions exert significant impact on students’ learning and achievement [1, 2]. Indeed, emotions can “control the students’ attention, influence their motivation to learn, modify the choice of learning strategies, and affect their self-regulation of learning” [3, p.6]. Some of the well-investigated emotions in education are different forms of anxiety such as test anxiety, math anxiety and general anxiety [4]. Test anxiety refers to the cognitive, behavioral, and physiological symptoms of anxiety associated with taking tests [5]. Math anxiety refers to the feelings of fear, tension, and apprehension accompanying learning math [6]. General anxiety refers to “feelings of nervousness, tenseness, or panic in reaction to diverse situations; frequent worry about the negative effects of past unpleasant experiences and future negative possibilities; feeling fearful and apprehensive about uncertainty; expecting the worst to happen” [7, p. 481]. In the current study, we investigated another form of educationally relevant anxieties–science anxiety that seriously impedes students’ learning and achievement [8].

### Science anxiety

Science anxiety is defined as “a debilitating combination of fearful negative emotion and cognition in the context of science learning” [9, p. 432]. Science anxiety can occur before or during learning science material [10, 11]. Many studies have consistently reported that math anxiety is a separate construct, which differs in some peculiar characteristics from other types of anxiety in education [e.g., for a review see 12]. Importantly, however, it is not clear whether science anxiety is a separate construct or a form of other types of anxiety in education [e.g., for a review see 11]. Interestingly, Bryant et al. (2013) used structural equation modelling (SEM) to study the association between science anxiety and the Constructivism Questionnaire’s three sub-scales (Negativity of Science toward the Individual, Subjective Construction of Knowledge, and Inherent Bias against Women) using samples of American and Danish participants [9]. Using the American sample, science anxiety was positively associated with Negativity of Science toward the Individual but not with Inherent Bias against Women and Subjective Construction of Knowledge. In contrast, using the Danish sample, science anxiety was positively associated with Subjective Construction of Knowledge but not with Inherent Bias against Women and Negativity of Science toward the Individual [9].

Students with high levels of science anxiety reported that they had ineffective high school science teachers, avoided science in college, and had lower SAT-Quantitative scores [13]. Mallow and colleagues [14–16] found that science anxiety was positively associated with non-science anxiety (which refers to students’ negative feelings towards other subjects) and choosing majors in humanities and social sciences. In addition, Ardasheva et al. (2018) found that students with higher science vocabulary knowledge had lower levels of science anxiety [17]. In addition, several studies reported negative associations between science anxiety and self-efficacy toward science in grade-5 students [18], middle school students [17, 19], and high school students [20, 21]. Consistently, science anxiety was found to be negatively associated with science achievement [19, 21, 22]. However, the causal relationship between science anxiety and science performance is not clear. Carey et al. (2016) suggested a bidirectional relationship between math anxiety and math performance so that both constructs can influence one another in a vicious cycle [12]. A similar reciprocal interaction might explain the relationship between science anxiety and science performance.

### Gender differences in science anxiety

Mixed results were reported regarding gender differences in science anxiety. On the one hand, female students were found to experience higher science anxiety than male students in middle school [17], high school [20] and university [14–16, 23]. Udo et al. (2004) suggested that males, based on cultural expectations, tend to under-report their level of science anxiety to meet with the stereotypic pressures on them to specialize in science and to deny the need for emotional support, whereas females tend to over-report their science anxiety levels [16]. On the other hand, Griggs et al. (2013) found a positive correlation between gender and science anxiety in fifth graders, indicating that boys had more science anxiety than girls, but there was no gender difference in self-efficacy toward science [18]. In contrast, some studies found no gender difference in science anxiety in primary school students [24] and university students [13], although females had higher science grades in high school than males [13]. Although some studies reported that science anxiety begins as early as age nine or grade 5 [18], to our knowledge, no studies have previously investigated the interaction between gender and grades in science anxiety [17, 18, 24].

### Scales to measure science anxiety

The measurement of science anxiety has received relatively less attention than other forms of anxiety in education such as test anxiety [e.g., for a review see 5] and math anxiety [e.g., for recent reviews see 25, 26]. To our knowledge, three scales have been originally constructed to measure science anxiety: the Science Anxiety Questionnaire (SAQ) [14], the Attitude Scale for Science and Technology (ASST) [26], and the Science Anxiety Scale (SAS) [27].

The SAQ [14] consists of 44 items measuring science anxiety (22 items) versus non-science anxiety (22 items) in university students. Each item is answered using a 5-point rating scale (not at all, a little, a fair amount, much, and very much), with high alpha Cronbach reliability (αs = .90 and .85 for science anxiety and non-science anxiety, respectively). However, the structure of the SAQ has not been investigated.

The ASST [24] has been constructed in Turkish and validated on middle school students. This scale consists of 21 items using a 5-point ranking scale. Using an exploratory factor analysis (EFA), Akpınara et al. (2009) found that the ASST involves four factors: Enjoyment of Science (8 items, α = .85), Anxiety of Science (7 items, α = .80), Interest in Science (3 items, α = .71), and Enjoyment of Science Experiments (3 items, α = .78) that accounted for 44.98% of the total variance.

Similarly, the SAS [27] has also been constructed in Turkish and validated on primary school students. This scale consists of 28 items using a 5-point rating scale. An initial exploratory factor analysis yielded two factors: Personal (23 items, α = .94) and Environmental (5 items, α = .77), which explained 54.11% and 5.33% of the total variance, respectively. A confirmatory factor analysis (CFA) supported the two-factor structure of this scale, with factor loadings ranged from .25 to .81 [27].

Other measures of science anxiety were adapted from questionnaires that were originally constructed to assess math anxiety, but their factorial validity has not been investigated. For example, Griggs et al. (2013) adapted the Math Anxiety subscale of the Student Beliefs about Mathematics survey [28] to assess science anxiety in fifth graders in the USA [18]. This adapted science anxiety scale consists of five items (e.g., “I’m usually calm during science tests”) using a 4-point rating scale, with a relatively low reliability rate (α = .62) [18]. In addition, Ardasheva et al. (2018) adapted the Mathematics Self-Efficacy and Anxiety Questionnaire [29] to assess science anxiety in eighth graders in the USA [17]. This adapted science anxiety scale consists of five items (e.g., “I worry that I will not be able to get a good grade in my science class”) using a 5-point rating scale, with an alpha reliability rate of .83 [17]. Furthermore, Britner (2008) adapted the Physiological States subscale of the Sources of math Self-Efficacy Scale [30] to assess science anxiety in high school students in the USA [20]. This adapted science anxiety scale consists of eight items (‘‘Science makes me feel uneasy and confused”) using a 5-point ranking scale, with a Cronbach’s alpha reliability rate of 0.92 (21).

### The current study

As discussed earlier, there are few measures of science anxiety including the SAQ [14], the ASST [24], and the SAS [27]. In addition, the psychometric properties of other science anxiety instruments that were previously adapted from different math anxiety questionnaires have not been yet investigated [17, 18, 20]. Furthermore, previous studies have investigated the associations among math anxiety, test anxiety, and general anxiety, suggesting that these three forms of anxieties represent distinct constructs, despite the modest positive correlations among them [31–35]. In agreement with these findings, modest positive associations between science anxiety and none-science (general) anxiety [14–16] have been previously reported. Importantly, however, no study has yet examined the associations among science anxiety, test anxiety, and general anxiety. Therefore, in the present study, we have developed the Abbreviated Science Anxiety Scale (ASAS), and examined the associations among science anxiety, test anxiety, and general anxiety.

The ASAS is adapted from the modified Abbreviated Math Anxiety Scale (m-AMAS) [31] to measure science anxiety in children and adolescents. Adjustments were made by replacing “math” environments with “science” contexts. For example, the original item stating “Thinking about a maths test the day before you take it” was modified to “Thinking about a science test the day before you take it”. However, face validity was not assessed. Among other scales of math anxiety [e.g., for reviews see 12, 25, 26], the m-AMAS has been selected for several reasons such as being brief, having adequate psychometric properties, and demonstrating good suitability for children and adolescents [12].

The m-AMAS was adapted for use with children from the Abbreviated Math Anxiety Scale (AMAS) [33]. The AMAS is a nine-item questionnaire, which requires participants to indicate how anxious they would feel during certain situations in math class using a 5-point Likert ranking scale, ranging from 1 (low anxiety) to 5 (high anxiety). This scale consists of two subscales: Learning Math Anxiety (5 items; e.g., “Listening to a lecture in math class”) and Math Evaluation Anxiety (4 items; e.g. “Taking an examination in a math course”); in addition to the total score representing a summation of the nine items. Using an exploratory factor analysis, Hopko et al. (2003) found that these two factors accounted for 70% of the total variance on the AMAS, with factor loadings ranging from .52 to .86 on LMA and from .66 to .89 on the MEA [33]. In addition, confirmatory factor analysis (CFA) supported this two-factor structure of the AMAS [33]. Further, Hopko et al. (2003) reported high internal consistency (αs = .90, .85, and .88) and test-retest reliability (*r*s = .85, .78, and .83) for the total score, LMA, and MEA, respectively [33]. The AMAS has been translated from English to different languages such as Italian [36], Polish [37], and Persian [38]. Across these translations, the two-factor model of the AMAS has been conformed, with adequate to strong psychometric properties.

Across two studies, the present paper examined the psychometric properties of the ASAS. Using samples of students from grades 7 to 10, Study 1 investigated the factorial structure of the ASAS along with its criterion validity with science achievement and convergent validity with test anxiety and general anxiety. In addition, the internal reliability of the ASAS factors, and gender and grade differences in science anxiety were investigated. Study 2 aimed to replicate the factorial structure of the ASAS and its associations with test anxiety and general anxiety using samples of students in grades 11 and 12. Based on preferences, high school students in most, if not all, Arab countries are divided into two main tracks: Sciences (which includes advanced chemistry, physics, biology, and math) and Arts (which include history, geography, psychology, sociology, and philosophy), more information can be found in the UNESO’s Global education monitoring report (2019) in Arab countries [39]. Therefore, Study 2 investigated whether science and art track students indeed show different science anxiety in line with previous conclusions that were made using university students [14–16].

## Study 1

### Method

#### Participants

A total of 710 students (372 females and 338 males) in grades 7 to 10 in four government schools (two preparatory and two secondary) in Qatar volunteered to participate in this study. Four classes in each grade (from 7 to 10) in the four schools were randomly selected. Table 1A shows some characteristics of this sample. Ethical approval for participation in the present two studies was provided by Qatar University’s institutional review board (QU-IRB) and all methods were administered in accordance with QU-IRB guidelines and regulations. Written informed consents were obtained from all participants and their parents before being included in the studies.

**Table 1:** Characteristics of participants in Study 1 and 2.

A. Study 1					
Grade		Grade 7	Grade 8	Grade 9	Grade 10
Girls	N	81	99	103	92
	Age (mean and SD in years)	12.2 (.5)	13.2 (.5)	14.1 (.5)	15.4 (.7)
Boys	N	83	68	85	99
	Age	12.6 (.7)	13.6 (.8)	14.4 (.8)	15.7 (.7)
B. Study 2					
Grade		Grade 11		Grade 12	
Track		Sciences	Arts	Sciences	Arts
Girls	N	46	47	41	51
	Age (mean and SD in years)	16.2 (.5)	16.4 (.7)	16.9 (.4)	17.1 (.6)
Boys	N	45	39	40	53
	Age	16.5 (.6)	17 (1.1)	17.3 (.7)	17.7 (.8)

#### Instruments

*(1) Science anxiety*. The Abbreviated Science Anxiety Scale (ASAS) has been adapted from the m-AMAS [31], which is, in turn, adapted from the AMAS [33] to measure science anxiety using a 5-point Likert ranking scale, ranging from 1 (low anxiety) to 5 (high anxiety). The ASAS consists of nine items, which belong to two main factors: LSA (5 items) and SEA (4 items). In addition, a total score represents the summation of these two factors. The ASAS is presented in the S1 Appendix.

*(2) Test anxiety*. The Brief FRIEDBEN Test Anxiety Scale [40] is a brief version of the FRIEDBEN Test Anxiety Scale, which measures test anxiety using a biopsychosocial model. Using exploratory factor analysis, von der Embse et al. (2013) extracted the highest loading 12 items belonging to three subscales: five items for social derogation, four items for cognitive obstruction, and three items for physiological tenseness [40]. Participants respond to each item using a six-point Likert ranking scale ranging from 1 (does not describe me at all) to 6 (describes me perfectly). CFA confirmed this three-factor structure, with Alpha Cronbach reliabilities of .88, .86 and .81 for social derogation, cognitive obstruction, and physiological tenseness, respectively [40].

*(3) General anxiety*. General anxiety was measured using the Personality Inventory for DSM-5 (PID-5) [7] sub-scale of Anxiousness, which consists of nine items. Each item requires participants to describe themselves using a 4-point Likert-type scale ranging from 0 (*very false or often false*) to 3 (*very true or often true*), and the scores reflect the average of responses. An Arabic translation for the PID-5 has been validated in Qatar, with a Cronbach’s Alpha reliability of .89 for this subscale [41, 42].

*(4) Science performance*. Consistent with previous studies [19, 21, 22], science achievement was measured using the students’ marks of science exams in the first semester. Marks of students in Grade 10 were calculated using the average marks across Biology, Physics, and Chemistry.

#### Translations & procedures

The m-AMAS [31] and test anxiety scale [40] were translated from English into Arabic with permission granted from the corresponding author of each questionnaire to the first author of the current study. Back-translation procedures were applied as following: (i) the scales were translated from English into Arabic; (ii) a professional translator, who had no prior experience with the scales, back-translated the Arabic versions into English; (iii) the back-translations were compared with the original scales by a native English researcher; (iv) modifications were made to produce the final Arabic translations of the scales.

The three scales of anxieties, ASAS, test anxiety, and general anxiety, were administered in groups during class attendance and all participants were encouraged to respond and not skip any item. The order of the scales was the same across all students, and adminstrations lasted for approximately 15 minutes. Notably, all data were collected during the second week of the second semester immediately after the reports of the first Semester were sent to all students.

#### Statistical procedures

Confirmatory Factor Analysis (CFA) was conducted using IBM Amos [43] to examine the factor structure of the ASAS. For evaluating the model fit, we used the common fit indices, including Comparative Fit Index (CFI; >.90), Tucker Lewis Index (>.90), Root mean square error of approximation (RMSEA; < .08), and Standardized Root Mean Square Residual (SRMR; < .08). In addition, CFA was used to examine how science anxiety was associated with test anxiety, general anxiety, and science achievement. McDonald’s ω was used to examine the internal reliabities of the ASAS and the other scales. To test the normality of science anxiety distributions, One-Sample Kolmogorov-Smirnov tests were performed. To examine gender differences across different grades, a series of 4 (grade) x 2 (gender) between-participant Analysis of Variances (ANOVAs) were performed. We report means, 95% confidence intervals, standard errors and standard deviations. In addition, Pearson correlation coeffients were used to examine the correlations among science anxiety, test anxiety, general anxiety, and science achievement. Finally, multiple linear regression analyses were conducted to a series of predictive models of science anxiety and science achievment.

### Results

#### Factor structure

**Fig 1:**
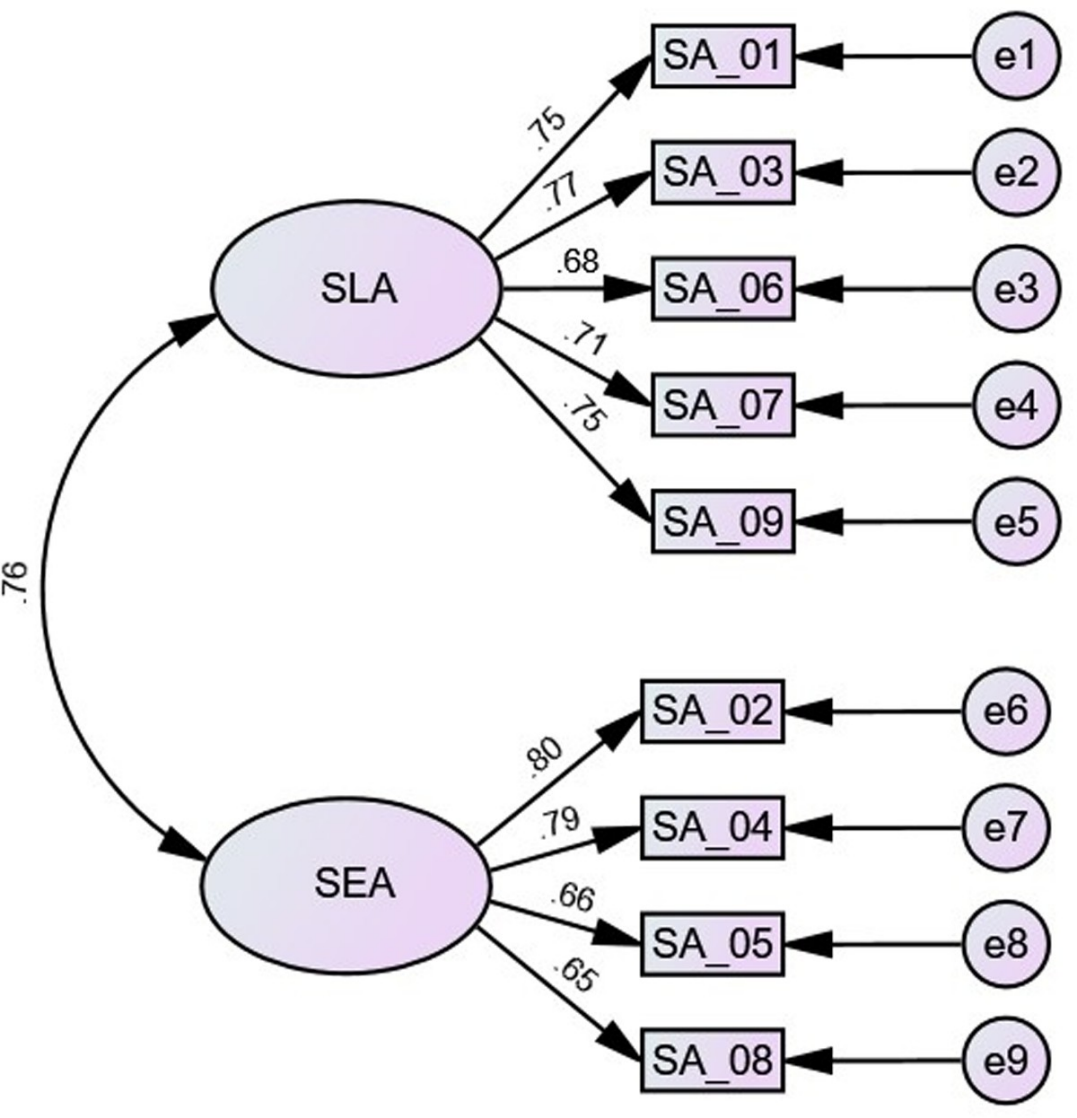
The factor structure of the ASAS in Study 1. SA = science anxiety; LSA learning science anxiety; SEA = science evaluation anxiety.

CFA supported the two-factor model of the ASAS, χ2 (26) = 153.649, p ≤ 0.001. Fit indices reported good to generally acceptable fit: CFI = .955; TLI = .938; SRMR = 0.075; and RMSEA = 0.083. In addition, the loadings of the two factors with their corresponding items were generally acceptable, ranging from .65 to .80, with means of .73 and .72 for the LSA and SEA; respectively (see Fig 1).

#### Internal reliability

Table 2 shows McDonald’s ω reliabilities of the scales. Good to adequate reliabilities were obtained for the three science anxiety scores across the four grades that ranged from .87 to .89 (for the total score), from .84 to .86 (for LSA), and from .77 to .83 (for SEA).

**Table 2:** McDonald’s ω reliability rates of anxiety scales in Study 1.

	Grade 7	Grade 8	Grade 9	Grade 10
science anxiety (9)	.87	.88	.89	.89
learning science anxiety (5)	.84	.84	.86	.86
science evaluation anxiety (4)	.77	.82	.83	.82
test anxiety (12)	.87	.87	.90	.91
social derogation (5)	.88	.82	.90	.91
cognitive obstruction (4)	.84	.87	.90	.91
physiological tenseness (3)	.81	.77	.86	.87
general anxiety (9)	.87	.81	.85	.85

#### Convergent and criterion validity

**Fig 2:**
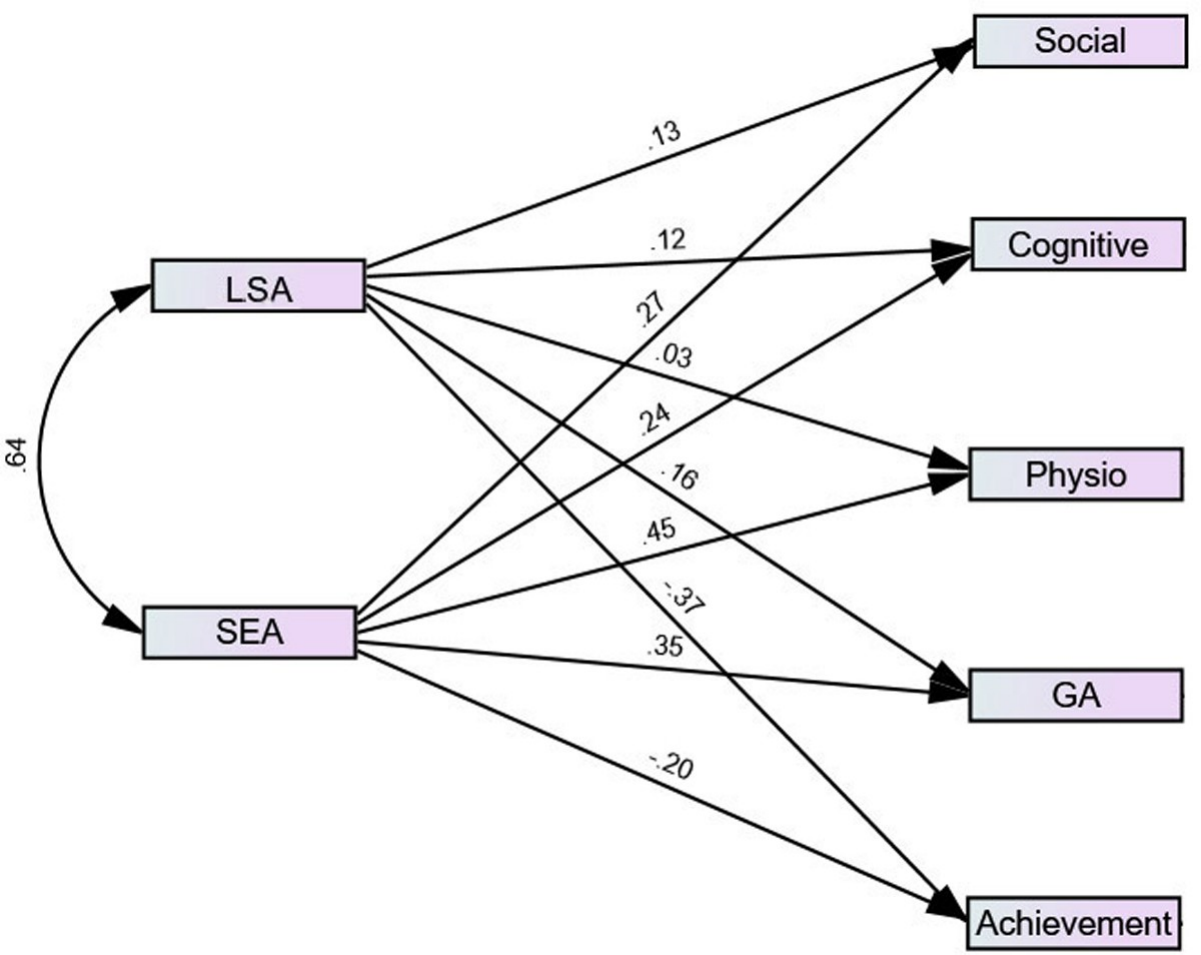
The associations between science anxiety subscales and test anxiety subscales, general anxiety, and science achievement. LSA = learning science anxiety; SEA = science evaluation anxiety; social = social derogation; cognitive = cognitive obstruction; Physio = physiological tenseness; GA = general anxiety; Achievement = Science Achievement.

Convergent validity was examined by the correlations between science anxiety and the other two types of anxiety, whereas criterion validity was indicated by the correlation between science anxiety and science achievements. As shown in Table 3, there were positive correlations between science anxiety and the other two types of anxiety, and the magnitudes of correlations with test and general anxieties were highly comparable with each other (mean *r*s = .42 and .43). In addition, there were strong negative correlations between science anxiety and science achievements (mean r = -.47). Fig 2 shows the standard loadings of science anxiety factors on test anxiety, general anxiety, and science achievement. The LSA was predicted only by science achievement (-.37) whereas SEA was predicted by all factors. However, CFA fit indices did not support this model (CFI = .779; SRMR = 3.306; RMSEA = .213; TLI = .536).

**Table 3:** The correlations among the three anxiety scales in Study 1.

	TA	Social	Cognitive	Physio.	GA	Achieve.
Grade 7						
SA	.39 (.25 –.53)	.32 (.15 –.47)	.28 (.14 –.43)	.37 (.22 –.50)	.49 (.34 –.61)	-.54 (-.68 –-.37)
LSA	.34 (.19 –.47)	.25 (.10 –.40)	.24 (.10 –.38)	.34 (.20 –.47)	.46 (.31 –.60)	-.53 (-.67 –-.36)
SEA	.38 (.22 –.53)	.33 (.14 –.49)	.28 (.13 –.42)	.33 (.18 –.47)	.42 (.27 –.55)	-.45 (-.59 –-.30)
Grade 8						
SA	.51 (.38 –.63)	.39 (.25 –.51)	.41 (.27 –.54)	.46 (.32 –.57)	.40 (.27 –.52)	-.42 (-.55 –-.28)
LSA	.39 (.26 –.53)	.31 (.17 –.45)	.33 (.19 –.48)	.30 (.16 –.44)	.32 (.18 –.46)	-.34 (-.48 –-.19)
SEA	.55 (.42 –.67)	.40 (.26 –.53)	.41 (.29 –.54)	.54 (.41 –.65)	.41 (.27 –.54)	-.44 (-.57 –-.30)
Grade 9						
SA	.34 (.20 –.50)	.25 (.11 –.41)	.20 (.06 –.34)	.34 (.20 –.47)	.33 (.19 –.47)	-.42 (-.56 –.25)
LSA	.32 (.18 –.48)	.25 (.11 –.41)	.20 (.07 –.33)	.27 (.14 –.40)	.26 (.13 –.41)	-.44 (-.58 –-.28)
SEA	.30 (.16 –.45)	.20 (.06 –.35)	.16 (.02 –.30)	.36 (.22 –.49)	.34 (.19 –.48)	-.31 (-.45 –-.15)
Grade 10						
SA	.56 (.47 –.67)	.50 (.37 –.61)	.39 (.26 –.52)	.52 (.42 –.61)	.58 (.48 –.68)	-.63 (-.71 –-.53)
LSA	.43 (.31 –.57)	.39 (.26 –.54)	.31 (.16 –.45)	.36 (.23 –.48)	.46 (.34 –.58)	-.62 (-.70 –-.53)
SEA	.60 (.51 –.69)	.50 (.38 –.61)	.41 (.27 –.53)	.58 (.48 –.67)	.60 (.49–70)	-.51 (-.62 –-.39)

*Note*: All ps ≤ 0.05; SA = science anxiety; LSA = learning science anxiety; SEA = science evaluation anxiety; TA = test anxiety; social = social derogation; cognitive = cognitive obstruction; Physio. = physiological tenseness; GA = general anxiety; Achieve. = Science Achievement.

#### Inter-correlations of the ASAS subscales

There were positive correlations between LSA and SEA in the four grades: Grade 7, *r* (164) = .66, p ≤ 0.001 (.52 –.75, 95% CI); Grade 8, *r* (165) = .67, p ≤ 0.001 (.57 –.76, 95% CI); Grade 9, *r* (186) = .64, p ≤ 0.001(.54 –.73, 95% CI); Grade 10, *r* (189) = .63, p ≤ 0.001 (.52 –.73, 95% CI).

#### Test score distributions

**Fig 3:**
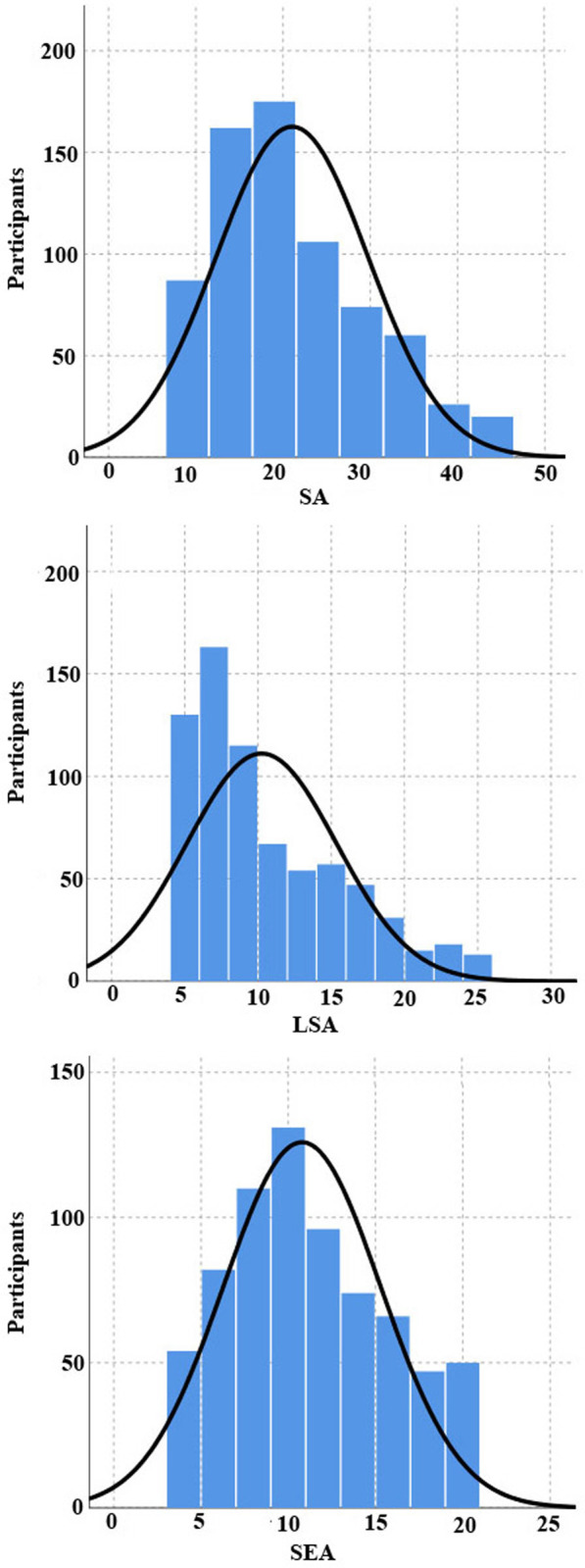
One-Sample Kolmogorov-Smirnov Tests for SA, SLA, and SEA in Study 1. SA = science anxiety; LSA = learning science anxiety; SEA = science evaluation anxiety.

Fig 3 shows the distributions of test scores. One-Sample Kolmogorov-Smirnov Tests showed that the distributions of SA, LSA, and SEA test results across the whole sample (*N* = 710) were normal, with means of 21, 10.2 and 10.8 (respectively) and standard deviations of 8.7, 5.1, and 4.5 (respectively), p’s (Lilliefors Corrected) ≤ 0.001. As shown in Fig 3, students’ responses ranged from 9 to 45 (for the total score), from 5 to 25 (for LSA), and from 4 to 20 (for SEA).

#### Gender and grade differences

**Fig 4:**
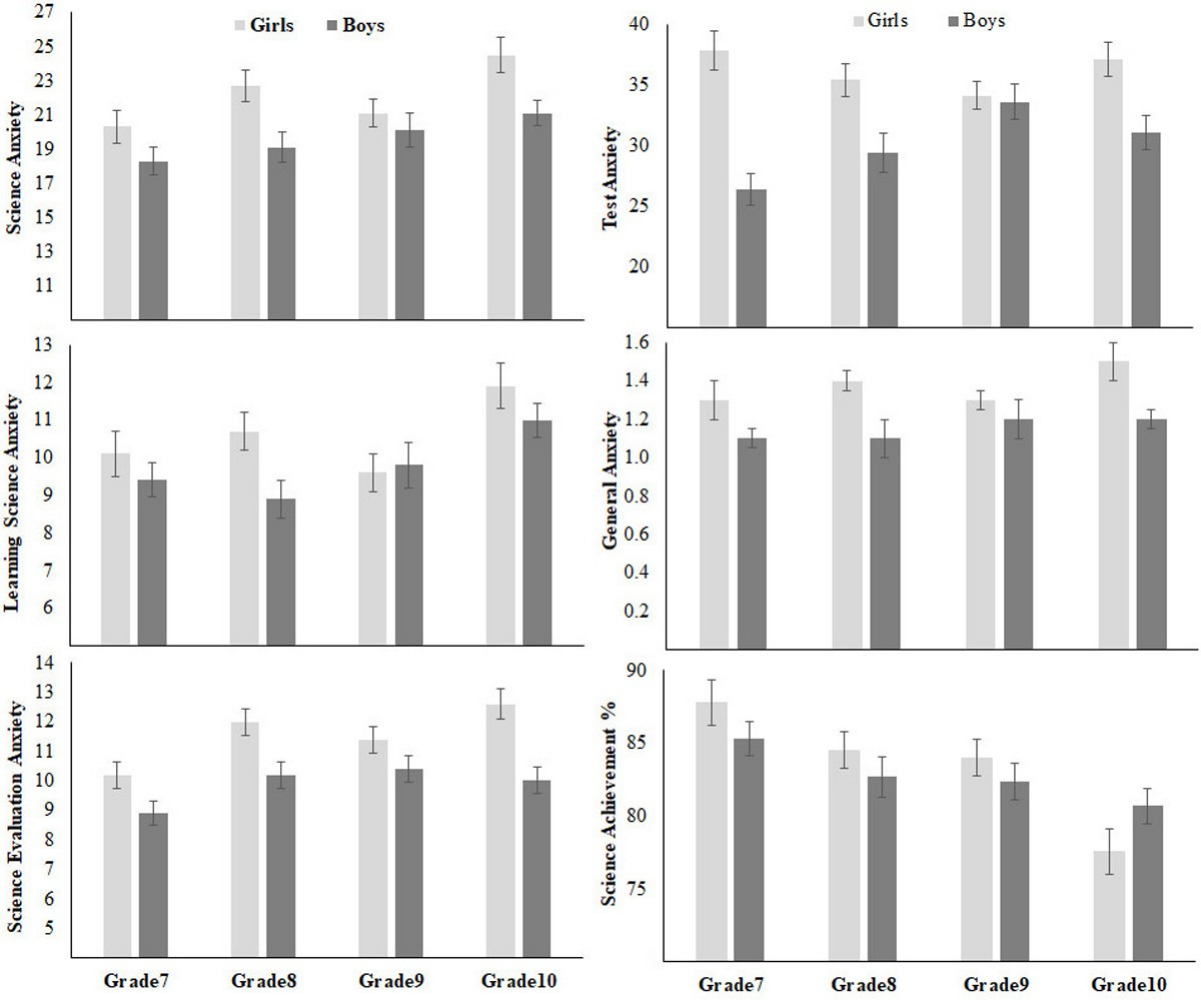
Gender and grade differences in Study 1.

Fig 4 shows means, with error bars representing standard errors, of students’ responses on the ASAS, test anxiety, general anxiety, and science achievement. Gender had main effects on SA, *F* (1, 702) = 15.23, p < 0.001, η_p_^2^ = 0.02, SLA, *F* (1, 702) = 4.70, p = 0.03, η_p_^2^ = 0.01, and SEA, *F* (1, 702) = 26.30, p < 0.001, η_p_^2^ = 0.04, showing higher science anxiety in females. In addition, grades had main effects on SA, *F* (3, 702) = 4.94, p = 0.002, η_p_^2^ = 0.02, SLA, *F* (3, 702) = 5.13, p = 0.002, η_p_^2^ = 0.02, and SEA, *F* (3, 702) = 5.70, p = 0.001, η_p_^2^ = 0.02. However, there was no interaction between gender and grades, *F*s ≤ 1. Post-hoc Scheffé test showed that grade 10 students had higher SA (mean = 22.7 ± 1.45; 95% CI) than grade 7 students (mean = 19.3± 1.3; 95% CI), *F* = 15.25. In addition, grade 10 students had higher LSA (mean = 11.5 ± .7; 95% CI) than students in grade 7 (mean = 9.7 ± .8; 95% CI) and grade 9 (mean = 9.7± .8; 95% CI), *F*s = 10.31 and 11.60; respectively. Furthermore, students in grades 8, 9, and 10 (respectively) had higher SEA (means = 11.3± .7, 10.9± .6 and 11.3 ± .7; 95% CIs) than students in grade 7 (mean = 9.5± .6; 95% CI). No other significant differences were found.

Gender also yielded main effects on test anxiety, *F* (1, 702) = 35.21, p ≤ 0.001, η_p_^2^ = 0.05, general anxiety, *F* (1, 702) = 14.63, p ≤ 0.001, η_p_^2^ = 0.02, and science achievement, *F* (1, 702) = 5.51, p ≤ 0.02, η_p_^2^ = 0.01, girls having higher scores on all of these measures. In addition, grades had a main effect on science achievement, *F* (3, 702) = 10.23, p ≤ 0.001, η_p_^2^ = 0.04, but not on test anxiety and general anxiety, *F*s <1. Once again, no interaction was found between gender and grades, all *F*s ≤ 1. Post-hoc Scheffé test showed that grade 10 students had lower achievement in science (mean = 79.1% ± 2%; 95% CI) than students in grades 7 (86.5% ± 1.9%; 95% CI), *F* = 30.92, p = 0.01, 8 (mean = 83.8% ± 1.9%; 95% CI), *F* = 12.54, p = 0.01, and 9 (83.3% ± 1.8%; 95% CI), F = 10.49, p = 0.01.

#### Multiple linear regression analysis

Multiple linear regression analyses were conducted to evaluate three models. Model 1 predicts science anxiety from gender, test anxiety, general anxiety, and science achievement using the whole sample. Model 2 predicts science achievement from science anxiety, test anxiety, and general anxiety in girls and boys separately. Model 3 predicts science achievement from learning science anxiety, science evaluation anxiety, test anxiety, and general anxiety in girls and boys separately. The results showed that all of these models were statistically significant: Model 1, SR, *F* (4, 709) = 132.29, p ≤ 0.001, R^2^ = 0.429, LSA, *F* (4, 709) = 89.67, p ≤ 0.001, R^2^ = 0.337, and SEA, *F* (4, 709) = 110.08, p ≤ 0.001, R^2^ = 0.384; Model 2, girls, *F* (3, 374) = 73.81, p ≤ 0.001, R^2^ = 0.374, and boys, *F* (3, 334) = 30.05, p ≤ 0.001, R^2^ = 0.214; Model 3, girls, *F* (3, 374) = 55.44, p ≤ 0.001, R^2^ = 0.375, and boys, *F* (3, 334) = 22.21, p ≤ 0.001, R^2^ = 0.217.

Table 4 shows the coefficients of these three models. To summarize, gender differences were statistically significant predictors of SA and SEA (but not LSA) even when test anxiety, general anxiety, and science achievement were included in the models (see Model 1 coefficients). In addition, SA correlated with science achievement in both girls and boys, even when TA and GA were considered (see Model 2 partial correlations). Furthermore, both SLA and SEA predicted science achievement in both girls and boys but the influence of SEA was stronger in girls than in boys (see standardized beta values in Model 3).

**Table 4:** The coefficients of the three regression models in Study 1. The coefficient of the multi regression model in Study 2.

Models	Standardized Beta	*t* (p)	Zero-order correlation	Partial correlation
Model 1				
SA				
Gender	-.102	-3.44 (p = 0.001)	-.142	-.128
Achieve.	-.404	-13.17 (p ≤ 0.001)	-.522	-.444
TA	.237	7.02 (p ≤ 0.001)	.466	.256
GA	.202	5.97 (p ≤ 0.001)	.462	.219
SLA				
Gender	-.050	-1.56 (p = .12)	-.072	-.059
Achieve.	-.407	-12.30 (p ≤ 0.001)	-.501	-.420
TA	.179	4.93 (p ≤ 0.001)	.378	.183
GA	.161	4.43 (p ≤ 0.001)	.386	.165
SEA				
Gender	-.140	-4.58 (p ≤ 0.001)	-.193	-.170
Achieve.	-.321	-10.08 (p ≤ 0.001)	-.442	-.355
TA	.255	7.29 (p ≤ 0.001)	.473	.265
GA	.207	5.92 (p ≤ 0.001)	.456	.218
Model 2				
Girls				
SA	-.533	-10.63 (p ≤ 0.001)	-.602	-.483
TA	-.007	-.13 (p = .90)	-.336	-.007
GA	-.125	-2.36 (p = .02)	-.408	-.122
Boys				
SA	-.415	-7.57 (p ≤ 0.001)	-.455	-.384
TA	-.038	-.68 (p = .49)	-.235	-.038
GA	-.071	-1.31 (p = .19)	-.230	-.072
Model 3				
Girls				
LSA	-.351	-6.43 (p ≤ 0.001)	-.559	-.317
SEA	-.231	-3.95 (p ≤ 0.001)	-.530	-.201
TA	-.009	-.17 (p = .86)	-.336	-.009
GA	-.130	-2.44 (p = .01)	-.408	-.126
Boys				
LSA	-.309	-4.79 (p ≤ 0.001)	-.438	-.255
SEA	-.143	-2.15 (p = .03)	-.384	-.117
TA	-.047	-.83 (p = .41)	-.235	-.046
GA	-.070	-1.29 (p = .20)	-.230	-.071
SA				
Gender	-.038	-.89 (p = .37)	-.094	-.047
Tracks	.316	7.34 (p≤ 0.001)	.398	.362
TA	.334	6.77 (p≤ 0.001)	.481	.337
GA	.179	3.64 (p≤ 0.001)	.396	.189
LSA				
Gender	.065	1.388 (p = .17)	.029	.073
Tracks	.361	7.683 (p≤ 0.001)	.409	.377
TA	.176	3.267 (p = .001)	.299	.170
GA	.136	2.539 (p.01)	.270	.133
SEA				
Gender	-.125	-2.915 (p = .004))	-.185	-.152
Tracks	.186	4.277 (p≤ 0.001)	.278	.221
TA	.393	7.885 (p≤ 0.001)	.523	.385
GA	.171	3.439 (p = .001)	.407	.179

*Note*: Data for gender factor was coded as 1 for girls and 2 for boys. Zero-order correlations refer to *r* (dependent, target variable), while partial correlations refer *r* (dependent, target variable) while partialling out the other variables. For gender factor, data was coded as 1 for girls and 2 for boys.

### Discussion

Converging with the factor structure of the AMAS [33] and the m-AMAS [31], the results of this study supported the two-factor structure of the ASAS (LSA and SEA; see Fig 1), with good internal reliability rates (see Table 1). In addition, there were strong negative correlations between science anxiety factors and science achievement (see Table 2), supporting the criterion validity of the ASAS. Further, there were positive associations between science anxiety factors and test anxiety and general anxiety. This finding converges with the results of previous studies reporting that math anxiety positively associated with test anxiety and general anxiety [32–35, 44]. Consistently, positive associations between science anxiety and none-science (general) anxiety has been previously found [14–16]. As shown in Fig 2, the LSA was predicted only by science achievement, whereas SEA was best predicted by physiological tenseness followed by general anxiety. Importantly however, CFA fit indices did not support this model, indicating that science anxiety might be a separate construct.

In agreement with previous studies [14–17, 20], girls showed higher science anxiety than boys, especially heightened anxiety related to science evaluation (SEA). Nevertheless, girls had higher marks in school science exams than boys. The higher female science anxiety score remained when test anxiety, general anxiety, and science achievement were considered in models. In addition, the results showed a main effect of grades on science anxiety, as students in Grade 10 were more anxious about science than those in lower grades. This might be related to the fact that science in Grade 10 is more detailed and specialized as students study separate courses for Biology, Physics, and Chemistry.

## Study 2

### Method

Study 2 had the following three main objectives: (i) replicate the factorial structure, reliability and inter-correlation of the ASAS in grade 11 and 12 students, (ii) replicate gender differences in science anxiety, and importantly (iii) investigate the differences in science anxiety between secondary school students who were in Sciences versus Arts tracks.

#### Participants

A total of 362 students (185 females and 177 males) in two government secondary schools in Qatar volunteered to participate in this study. Table 1B shows main descriptions of this sample. Data were collected with approval from the Ministry of Education and Higher Education in Qatar. In addition, eithical approval was obtained from the Qatar University IRB committee.

#### Instruments

The same instruments of Study 1 were ultizied in this study. However, no data for achievement were collected as students in Arts track did not take science courses.

#### Statistical analyses

CFA was conducted using IBM Amos [43] to replicate the two-factor structure of the ASAS using the same fit indices as used in Study 1. McDonald’s ω and Pearson Correlations with 95% confidence intervals were used to examine the reliability of the two factors and their inter-correlation, respectively. To examine the differences of science anxiety across genders, tracks, and grades, a series of 2 (gender) x 2 (track) x 2 (grades) between-participant ANOVAs were carried out. However, as grades have not shown main effects, a series of 2 (gender) x 2 (track) between-participant ANOVAs were conducted in order to simplify the analysis. Descriptive statistics, means along with SE, are presented. Finally, multiple linear regression analysis was conducted to examine how science anxiety could be predicted using gender, tracks, test anxiety, and general anxiety.

### Results

#### Factor structure

**Fig 5:**
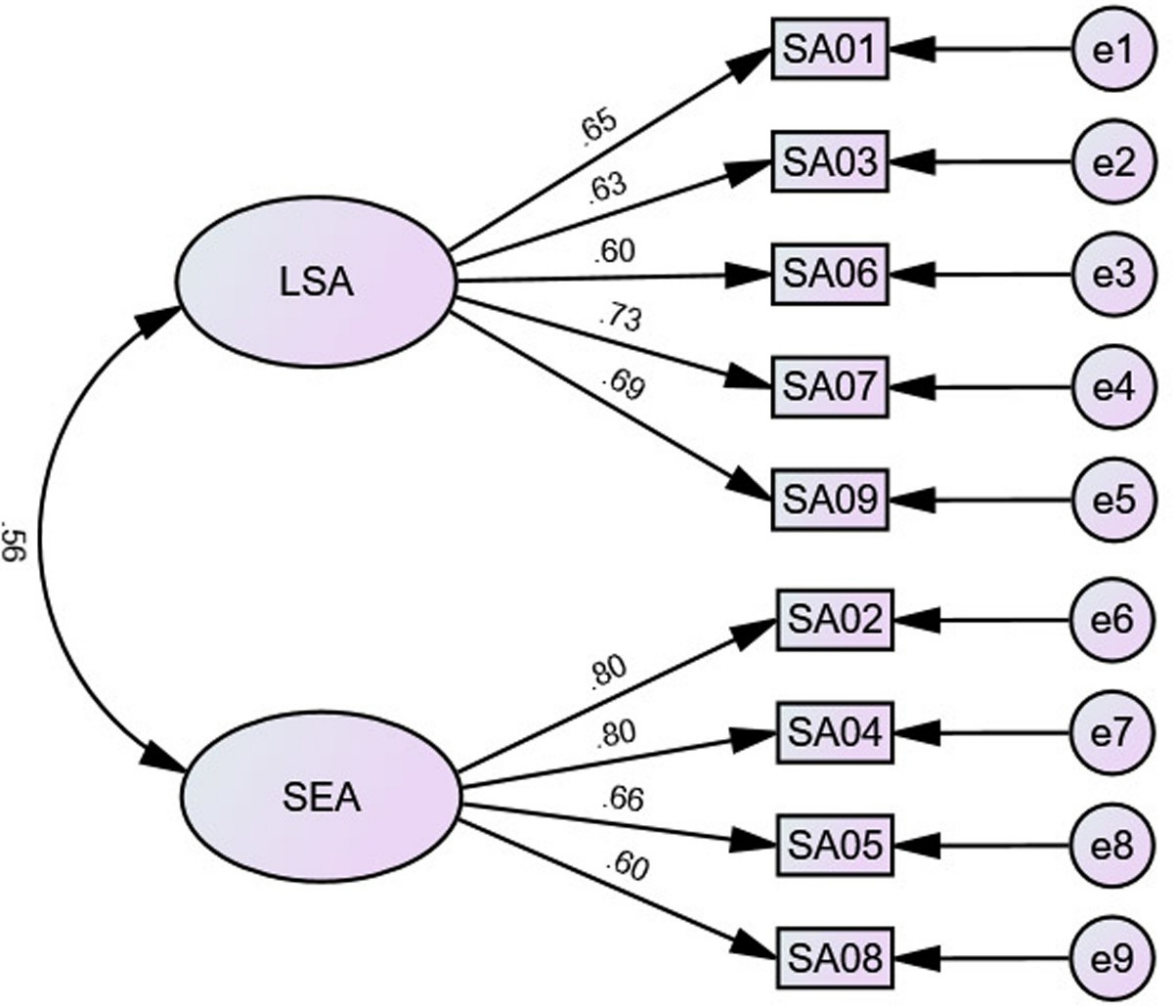
The factor structure of the ASAS in Study 2. LSA = learning science anxiety; SEA = science evaluation anxiety.

CFA supported the two-factor model of the ASAS, χ2 (26) = 70.527, p = < 0.001. Fit indices reported good to generally acceptable fit: CFI = .958; TLI = .941; SRMR = 0.073; and RMSEA = 0.069. In addition, the loadings of the two factors with their corresponding items were generally acceptable, ranging from .60 to .80, with means of .66 and .71 for LSA and SEA factors; respectively (see Fig 5).

#### Reliability and inter-correlation

Acceptable to adequate reliability rates were observed as follow: SA (ω = .84), LSA (ω = .79), and SEA (ω = .81). In addition, there were positive correlation between LSA and SEA, r (362) = .47, p < 0.001, 95% CI ranged from .40 to .55).

#### Gender and track differences

**Fig 6:**
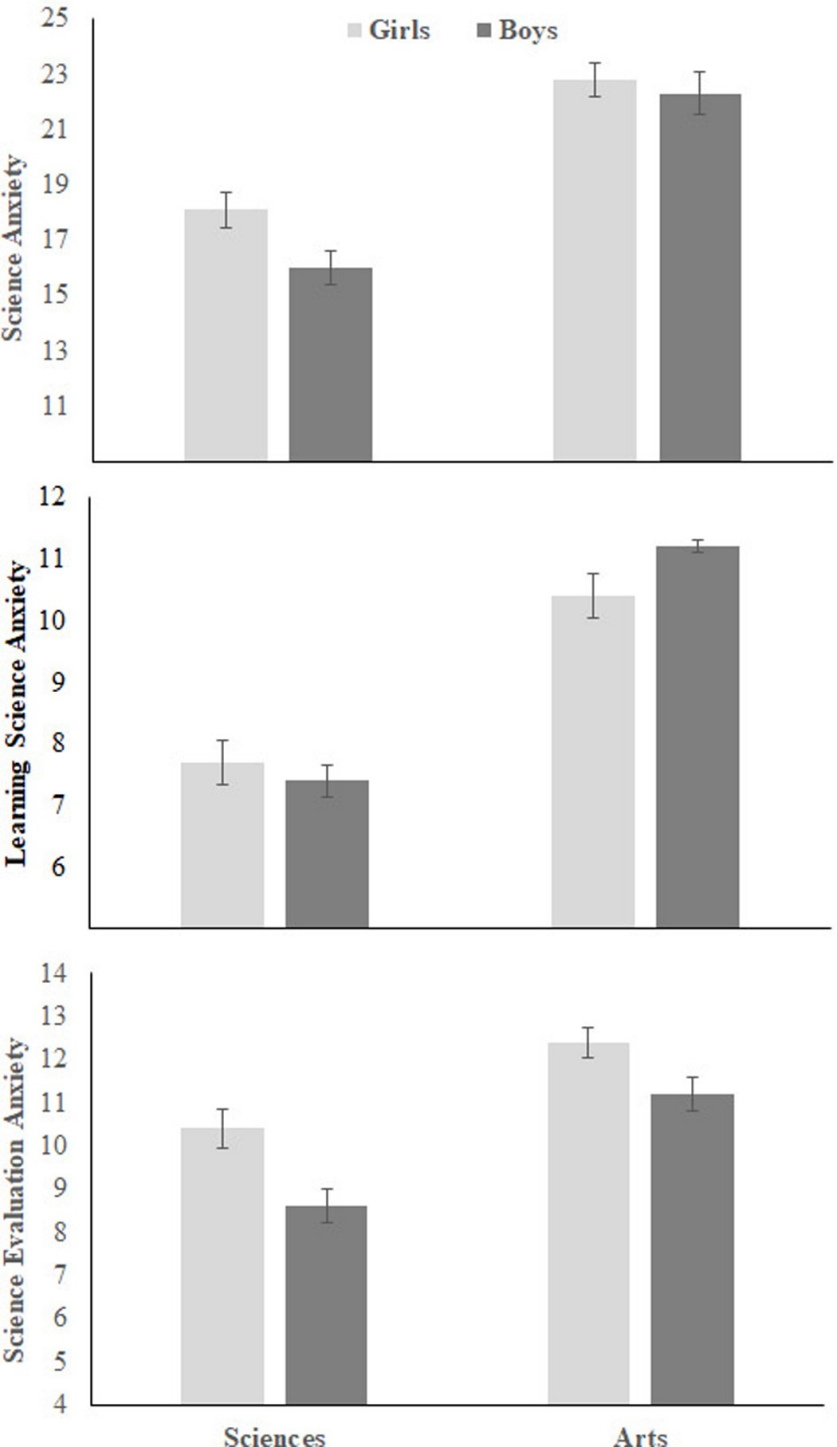
Gender and track differences in science anxiety in Study 2. The horizontal axis represents the science and arts tracks.

Fig 6 shows means, with error bars representing standard errors, of students’ responses on the ASAS. Females had higher SA and SEA scores than males but there was no significant difference in LSA: a series of 2 x 2 between-participant ANOVAs showed main effects of gender on SA, *F* (1,358) = 3.71, p = 0.054, η_p_^2^ = 0.01, and SEA, *F* (1,358) = 13.61, p ≤ 0.001, η_p_^2^ = 0.04, but not on LSA, F< 1. In addition, Arts Track students had higher scores on SA, LSA and SEA than Science Track students: there were Track main effects on SA, *F* (1,358) = 68.51, p ≤ 0.001, η_p_^2^ = 0.02, LSA, *F* (1,358) = 73.23, p ≤ 0.001, η_p_^2^ = 0.02, and SEA, *F* (1,358) = 30.80, p ≤ 0.001, η_p_^2^ = 0.04. There was no Gender × Track interaction, *F*< 1.

**Fig 7:**
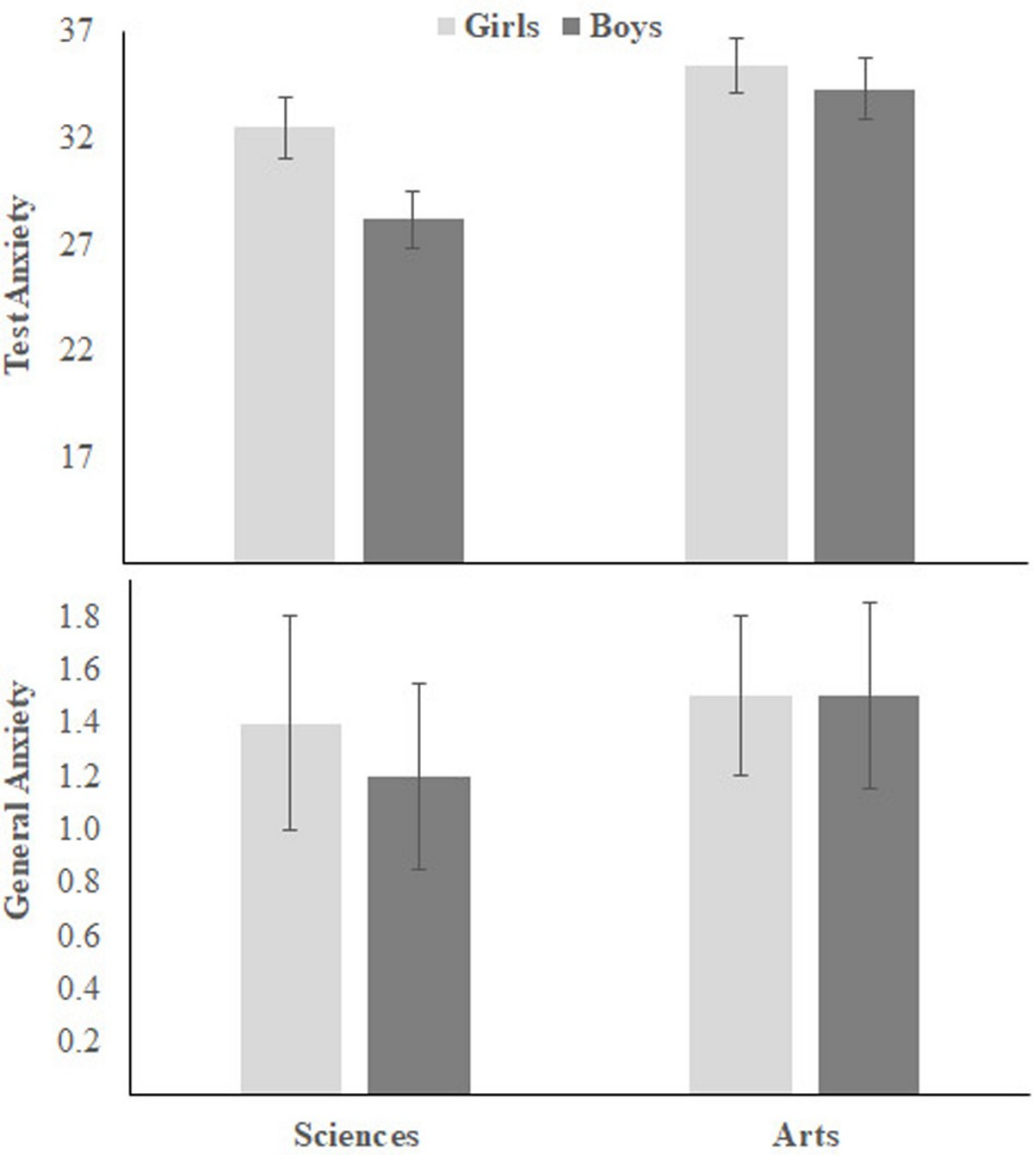
Gender and track differences in test anxiety and general anxiety in Study 2.

Fig 7 shows means, with error bars representing standard errors, of students’ scores on test anxiety and general anxiety. Female and Arts Track students had higher TA than male and Science Track students. Gender and tracks main effects on TA: *F*s (1,358) = 4.04 and 10.67, ps = 0.04 and 0.001, η_p_^2^ = 0.01 and 0.03 (respectively). Similarly, female and Arts Track students had higher GA than male and Science Track students. Gender and tracks main effects on GA: *F*s (1,358) = 3.92 and 7.61, ps = 0.04 and 0.006, η_p_^2^ = 0.01 and 0.02 (respectively). There was no Gender × Track interaction, *F*s ≤ 1.

#### Multiple linear regression analysis

Three multiple linear regression analyses evaluated a model using science anxiety scores (SA, LSA and SEA) as dependent variables and gender, Science Track, test anxiety, and general anxiety as predictors. Table 4 shows analysis outcomes. The model was statistically significant considering SA, *F* (4, 361) = 50.38, p ≤ 0.001, R^2^ = 0.361, LSA, *F* (4, 361) = 28.01, p ≤ 0.001, R^2^ = 0.239, and SEA, *F* (4, 361) = 47.93, p ≤ 0.001, R^2^ = 0.349. In summary, Gender was a significant predictor only for SEA. In contrast, Science Track was a significant predictor of SA, LSA, SEA.

### Discussion

The findings of the Study 2 replicated the results of Study 1 and confirmed the two-factor structure of the ASAS using student in grades 11 and 12, with good reliability rates for the two factors, which were positively correlated with each other. We also replicated gender differences as girls had higher science anxiety, especially SEA. In addition to the factorial validity, the present study provided good evidence for the discriminative validity of the ASAS as students in the Arts track had higher science anxiety than those in science track. This finding persisted even when gender, test anxiety and general anxiety were considered in models.

## General discussion

Across two studies, we investigated the psychometric properties of the ASAS, which was adapted from the m-AMAS [31]. Using a sample of students in grades 7 to 10, Study 1 reported a two-factor structure of the ASAS and strong negative associations between science anxiety and science achievement. Using a sample of students in grades 11 and 12, Study 2 replicated this two-factor model and additionally found that students in the Arts track were more anxious about science than students in the Sciences track. In addition, both studies reported good reliabilities for the ASAS factors and modest meaningful correlations with test anxiety and general anxiety suggesting that science anxiety might be a distinct construct. Furthermore, girls had higher SA (especially SEA) than boys, even when we controlled for test anxiety and general anxiety.

### The psychometric properties of the ASAS

The results of this study indicate adequate psychometric properties of the ASAS. For example, the two-factor model of the ASAS, with positive inter-correlations and good reliability rates. In addition, the ASAS scores correlated negatively with science achievement and positively with test anxiety and general anxiety. Therefore, the ASAS is a valid instrument, which can be used to measure two basis components of science anxiety (SLA and SEA) in students at middle and high schools. In fact, few science anxiety scales were previously developed. The SAQ consists of two subscales (science anxiety and non-science anxiety) [14]. The ASST involves four factors (enjoyment of science, anxiety of science, interest in science, and enjoyment of science experiments) [24]. The SAS has two factor (personal and environmental) [27]. Few science anxiety instruments were previously adapted from different math anxiety questionnaires, but their psychometric properties have not been yet investigated [17, 18, 20]. The ASAS has been adapted from the m-AMAS [31], which is, in turn, adapted for use with children from the AMAS [33]. The ASAS is a brief, valid, and reliable instrument that measure students’ negative emotions toward two fundamental elements of science education: learning and evaluation. Therefore, the ASAS could have significant implications for understanding and improving science education. In addition, it provides a unique opportunity for future studies to investigate the relationship between math anxiety and science anxiety without a need to use two very different scales for each construct.

### The relationship between science anxiety and science achievement

There were significant negative associations between science anxiety and science achievement (see Table 3 and Fig 2). In addition, the ASAS scores significantly predicted science achievement in both girls and boys (see Table 4). Furthermore, when controlling for test anxiety and general anxiety, there were significant negative partial correlations between the ASAS scores and science achievement (see Models 2 and 3 in Table 4). Consistently, previous studies reported that science anxiety was negatively associated with science achievement [19, 21] and self-efficacy toward science [17–21]. Importantly, however, the direction of these relationship is not clear as poor science performance might elicit science anxiety; science anxiety might lead to poor science achievement; or science anxiety and science achievement might reciprocally interact with each other. Notably, this same “chicken-egg” question has been debated for math anxiety [e.g., for a review see 12].

### Science anxiety as a separate construct

Science anxiety positively correlated with TA and GA (see Table 3). However, fit indices did not support a CFA model involving the ASAS subscales, TA, GA, and science achievement. As illustrated in Fig 2, LSA was modestly associated with science achievement and weakly associated with test anxiety and general anxiety. On the other hand, SEA was modestly associated with science achievement, test anxiety and general anxiety. Across the ASAS scores, the results showed that the predictors of science anxiety were, respectively, science achievement, test anxiety, general anxiety, and gender (see Table 4). When controlling for gender, general anxiety, and science achievement in the correlations between ASAS scores and test anxiety, there were significant positive partial correlation between ASAS scores and test anxiety. In addition, when controlling for gender, test anxiety, and science achievement in the correlations between ASAS scores and general anxiety, there were significant positive partial correlation between ASAS scores and general anxiety. Together, these modest associations among science anxiety, test anxiety, and general anxiety, suggesting that they are distinct constructs. Notably, this is the same approach, by which Hill et al. (2016) suggested that math anxiety is a distinct construct [45].

### Gender differences

Consistent with previous results [14–17, 20], the present study showed that girls had higher overall score on the ASAS than boys (see Figs 4 and 6). Girls had higher SEA than boys, but both groups had a similar level of LSA. However, consistent with previous studies (e.g., Brownlow et al., 2000), science achievement was higher in girls than in boys (see Fig 4). In addition, girls had higher TA and GA than boys (e.g., see Fig 4). When controlling for science achievement, TA and GA, girls still had higher SA and SEA than boys (see partial correlations in Table 4).

### Cultural aspects

Notably, the vast majority of psychological studies have been conducted with participants from western, educated, industrialized, rich and democratic (WEIRD) societies (mostly Americans [46, 47]. Similarly, most knowledge of emotions in classrooms have come from such WEIRD countries [e.g., for reviews see 1–5]. Previous studies have reported both cross-cultural similarities and variations in classroom emotions [48, 49]. The present study contributes novel data to this question from an Arabic speaking Middle Eastern culture.

## Limitations and conclusions

This study is not without limitations. The first limitation is not including the original measure of math anxiety (m-AMAS) [31]. Second, all participants in the present study were students in grades 7 to 12. Third, our study did not involve the use of school assessments for measuring science achievements. Fourth, all instruments were self-reported questionnaires. Finally, group differences across gender and grade were examined using inferential statistics (ANOVA), which might be sensitive to mean score differences between groups. Therefore, future studies should investigate the joint factorial structure of the ASAS and m-AMAS, the psychometric properties of the ASAS in primary and post-secondary education, the association between science anxiety and a standardized science achievement test, and the criterion validity of the ASAS with physiological measures of test and general anxieties. In addition, in order to make meaningful interpretations of group differences across gender and grade, future studies must establish measurement invariance to allow for a more conceptual-level comparison that should not be sensitive to mean score differences between groups [50]. The present study only presented some quantitative indications on those group differences. Despite these limitations, we have created a brief, valid, and reliable instrument for measuring students’ emotions toward two fundamental elements of science education, science learning and science evaluation. We also provided evidence that science anxiety is a distinct construct and observed marked gender differences in science anxiety.

## Supporting information

S1 Data Data for the two studies reported in this study. (XLSX)

S1 Appendix (DOCX)
