# Supplementary material for: The Abbreviated Science Anxiety Scale: Psychometric properties, gender differences and associations with test anxiety, general anxiety and science achievement
Source: PLoS One. 2021 Feb 12;16(2):e0245200. doi: 10.1371/journal.pone.0245200 (PMC7880483; doi:10.1371/journal.pone.0245200)
Supplement: S1 Appendix — (DOCX) [file pone.0245200.s002.docx]

**S1 Appendix**

**The Abbreviated Science Anxiety Scale**

Imagine yourself in the situations described below. Evaluate each situation in terms of how much fear you feel during the specified activities, putting a tick in the column that corresponds to your level of fear: please rate your feelings on a scale from one (no bad or negative feelings) to five (the worst feelings: the most fear, worry, or nervousness).

1 = No bad feelings; 2 = Somewhat bad; 3 = Moderate fearful, tense or nervous; 4 = Bad feelings; 5 = Very bad feelings.

|  | 1 | 2 | 3 | 4 | 5 |
| --- | --- | --- | --- | --- | --- |
| Having to complete a worksheet in science by yourself |  |  |  |  |  |
| Thinking about a science test the day before you take it |  |  |  |  |  |
| Watching the teacher work out a science experiment on the board |  |  |  |  |  |
| Taking a science test |  |  |  |  |  |
| Being given science homework with lots of difficult questions that you have to hand in the next day |  |  |  |  |  |
| Listening to the teacher talk for a long time in science |  |  |  |  |  |
| Listening to another child in your class explain a science problem |  |  |  |  |  |
| Finding out that you are going to have a surprise science quiz when you start your science lesson |  |  |  |  |  |
| Starting a new topic in science |  |  |  |  |  |
